# A rare presentation of visceral leishmaniasis and epididymo‐orchitis in a patient with chronic granulomatous disease

**DOI:** 10.1002/ccr3.7426

**Published:** 2023-05-31

**Authors:** Shaghayegh Ashraf Talesh, Shima Mahmoudi, Mehdi Mohebali, Setareh Mamishi

**Affiliations:** ^1^ Department of Infectious Diseases, Children's Medical Center Tehran University of Medical Sciences Tehran Iran; ^2^ Pediatric Infectious Disease Research Center Tehran University of Medical Sciences Tehran Iran; ^3^ Department of Parasitology and Mycology, School of Public Health Tehran University of Medical Sciences Tehran Iran

**Keywords:** chronic granulomatous disease, epididymo‐orchitis, visceral leishmaniasis

## Abstract

Visceral leishmaniasis (VL) has rarely been reported in patients with chronic granulomatous disease (CGD), despite the fact that they seem more susceptible to intracellular infection. We describe a rare presentation of VL and epididymo‐orchitis in a patient with CGD, which has not been seen previously in the literature following inflammatory syndrome.

## Graphical abstract

Leishman's body was observed in a bone marrow sample of a 19‐year‐old man with chronic granulomatous disease (CGD) and epididymo‐orchitis.

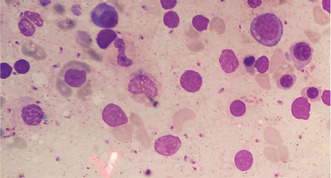

## 1 INTRODUCTION

Chronic granulomatous disease (CGD) is a condition caused by immunodeficiency problems in phagocytes that leads to dysfunctional phagocytes incapable of killing a variety of bacteria and fungi.^1^ CGD disorder is caused by defects in any of the five subunits of the nicotinamide adenine dinucleotide phosphate (NADPH) oxidase complex. This complex is responsible for respiratory bursts in phagocytic leukocytes.^2^

Catalase‐positive bacteria and fungus infections can pose a life‐threatening threat to patients with CGD.^3^ There have been rare reports of visceral leishmaniasis (VL) in CGD cases, despite the fact that they seem more susceptible to intracellular infection.^4^ Leishmaniosis can be cutaneous, mucocutaneous, or visceral (kala‐azar is the most severe form).^5^ Kala‐azar, or VL, is a deadly disease transmitted by female phlebotomine sand flies carrying over 20 species of leishmania.^6^ In some areas of Iran (northwest and southeast), leishmaniasis is endemic.^7^ Symptoms and signs associated with VL, include fever, chills, fatigue, weight loss, loss of appetite, hepatosplenomegaly, and lymphadenopathy.^8^ In this case report, an unusual combination of epididymo‐orchitis and VL in a CGD patient is presented, and we discuss the challenges of treating leishmaniasis.

## 2 CASE PRESENTATION

**FIGURE 1:**
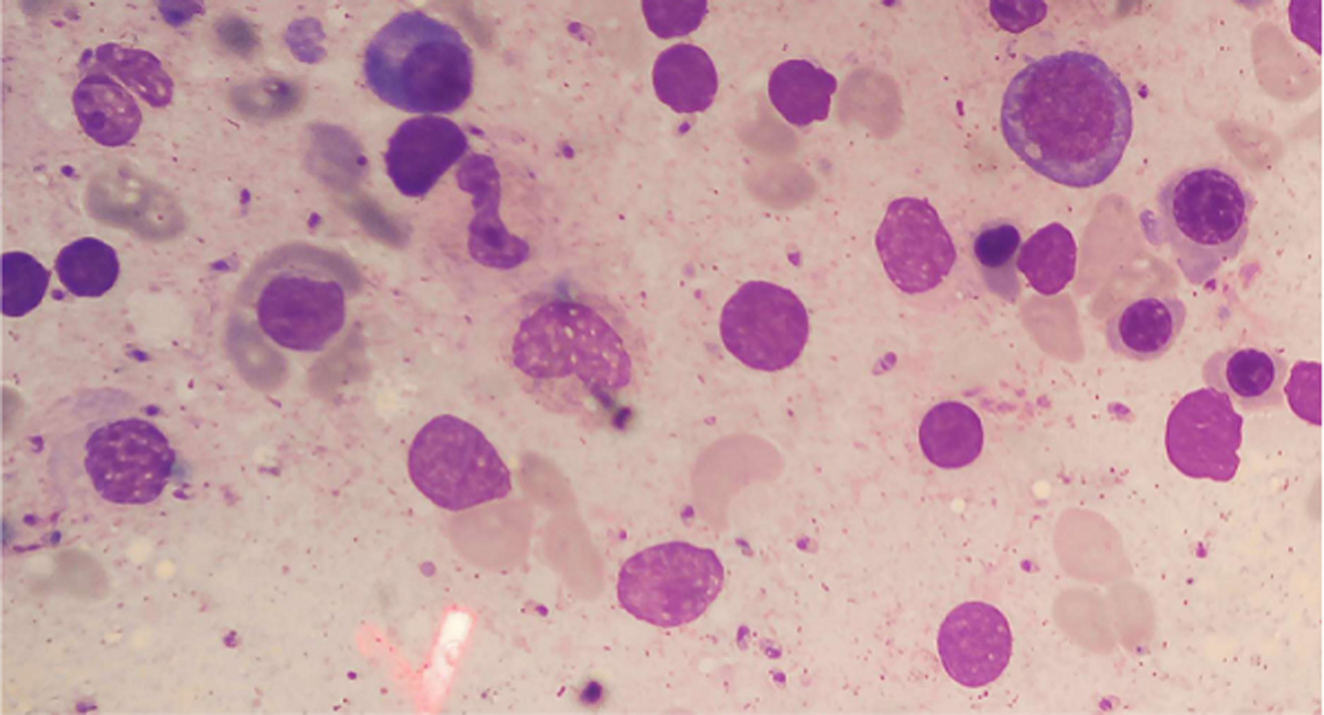
The bone marrow aspirate shows leishmaniasis with Wright‐Giemsa stain.

A 19‐year‐old man with Chronic granulomatous disease (CGD) diagnosed at 6 months of age presented to the infectious disease ward with fever, orchitis, sweating, anorexia, and a weight loss of eight kilograms. Over the last few days, the fever has increased, and chills have been present as well. Outpatient treatment with acetaminophen and levofloxacin resulted in no improvement. The patient was toxic and pale upon entering the hospital. He has not been using chemoprophylaxis drugs for CGD. In the examination, there was swelling of the left testis, tenderness in the abdomen, and a palpable spleen (7 cm below the costal margins). Following complaints of testicular pain and swelling, an ultrasound was performed, which revealed that there was a small volume of hydrocele on the left side, along with debris in the hydrocele fluid, in favor of epididymitis. On arrival at the hospital, his vital signs were as follows: blood pressure of 100/60 mmHg, heart rate of 100/min, temperature of 39 degrees Celsius, and respiratory rate of 30/min. Initial laboratory tests were done: a white blood cell count of (WBC) = 1.5 × 10^6^/uL; c‐reactive protein (CRP) = 35 mg/L; hemoglobin (Hb) = 7/7 g/dL; platelets 125 × 10^6^/uL; erythrocyte sedimentation rate (ESR) = 94 mm/h (Table 1). Wright and 2ME (2‐mercaptoethanol) were negative. He was treated experimentally with ceftazidime and vancomycin because of his toxic condition. Abdominal sonography was performed and massive splenomegaly was reported. Because of the patient's toxic condition, persistent symptoms, pancytopenia, and travel history to Ardabil, one of the country's endemic areas for leishmania, he was empirically treated with amphotericin liposomal (5 mg/kg per day). A bone marrow sample was also taken to rule out malignancy. No evidence of malignancy was found in the bone marrow sample, which revealed the presence of Leishman's body (Figure 1).

**TABLE 1:** Summary of clinical investigations during hospitalization.

Laboratory test	Value at admission	Value after 7 days	Normal range
WBC count (×10^6^/uL)	1.5	1.25	4–10
Platelet (×10^6^/uL)	125	110	150–450
Hb (g/dL)	7.7	4.7	11–16
CRP (mg/L)	35	135	0–6
ESR (mm/h)	94	125	0–10
Fibrinogen (mg/dL)	–	320	150–350
Ferritin (ug/L)	–	1320	30–220
D‐dimer (ng/mL)	–	1057	<250
Brucella serology	Negative		
Direct Agglutination Test	Positive		
K39 Test	Positive		
Immunofluorescence assay	Positive		

Abbreviations: CRP, c‐reactive protein; ESR, erythrocyte sedimentation rate; Hb, hemoglobin; WBC, white blood cell.

**FIGURE 2:**
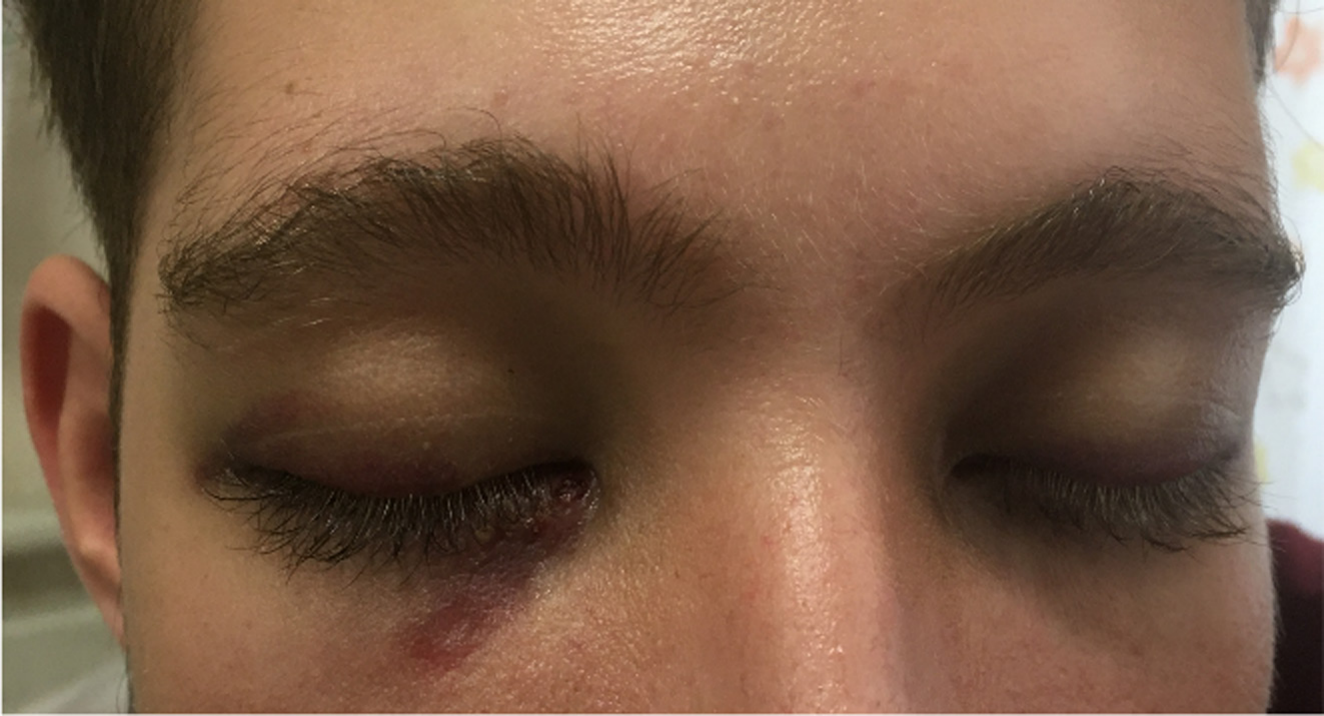
Erythematous patches were observed above the eyelids, under the eyes.

Consequently, amphotericin treatment continued. The K39 test and the Direct Agglutination Test (DAT) were performed. The test results were as follows: DAT = 1/102400 (>1/3200 is considered positive), Immunofluorescence assay (IFA) positive, and K39 positive. Bone marrow culture revealed *Staphylococcus epidermidis* which was sensitive to vancomycin. Throughout the treatment, erythematous patches were observed above the eyelids and under the eyes, and to a lesser extent on the trunk, an investigation of cutaneous leishmaniosis was performed (Figure 2). Dermatophyte lesions were found on a skin smear, while no Leishman's body could be found.

During hospitalization, the platelet and neutrophil counts decreased. As a result of the decrease in platelets, transfusions were administered, and for persistent neutropenia a granulocyte colony‐stimulating factor G‐CSF 300 mcg for 3 days was prescribed. As the fever did not respond to broad‐spectrum antibiotic therapy, the authors suggest a Jarisch‐Herxheimer reaction to account for this strange outcome; therefore, it was decided to begin oral treatment with prednisolone (2 mg/kg/day). Finally, the fever subsided after 2 weeks of treatment, and the general condition improved. With the improvement of his clinical conditions, he was discharged. To maintain drug therapy at home, glucantime was administered every 12 h intramuscularly (20 mg/kg of pentavalent antimony, which is equal to 75 mg/kg of glucantime), and prednisolone (2 mg/kg/day). But 1 week later, the patient was readmitted to the hospital with severe anemia, leukopenia, and orchitis. Despite the blood injection, hemoglobin did not increase. Nothing was found in the workup for sepsis. Laboratory tests showed: WBC = 1.250 × 10^6^/μL, Hb = 4.7 g/dL, platelets = 110 × 10^6^/μL, ESR = 125 mm/h, CRP = 135 mg/L, D‐dimer = 1057 ng/mL, ferritin = 1320 ng/L, and fibrinogen = 320 mg/dL (Table 1). An inflammatory syndrome was deemed to be present based on high inflammatory elements and a negative sepsis workup. Intravenous immunoglobulin (IVIG, 1gr/kg) for 2 days along with methylprednisolone (1 mg/kg/day) was prescribed. The patient's HB levels rose after 4 days, and the fever stopped, resulting in an improvement in general health. The tests on the day of discharge were as follows: WBC = 1.79 × 10^6^/uL, Hb = 8.3 g/dL, platelets = 214 × 10^6^/uL. In 6 months of follow up his condition was good.

## 3 DISCUSSION

CGD is a condition caused by immunodeficiency problems in phagocytes that leads to dysfunctional phagocytes incapable of killing a variety of bacteria and fungi.^1^ VL has rarely been reported in patients with CGD, despite the fact that they seem more susceptible to intracellular infection.^7^

To our knowledge, this is the first reported case of a combination of epididymo‐orchitis. Finocchi et al. reported the first case of VL infection in a 3‐year‐old child with CGD.^9^ Further, Al Ayed et al. reported the case of an infant who had disseminated L. *donovani* and CGD Bogdan et al. described an unusual case of VL caused by *L. infantum* occurring in a 15‐month‐old German child who had never lived in an area of endemic disease and who had no other sources of infection (e.g., blood transfusion or congenital).^10^

This report describes a nineteen‐year‐old man who was admitted with a fever for 1 month, orchitis, sweating, anorexia, and a weight loss of eight kilograms. The patient had a fever, pancytopenia, massive splenomegaly, and orchitis, which prompted us to run an endemic disease workup on him. Wright and 2ME were requested, and the results were negative. In spite of the fact that viruses are the most commonly reported causes of orchitis, in this case, the patient's condition was septic and he was immune deficient, so he was experimentally treated with ceftazidime and vancomycin.^11^ In some areas of Iran (northwest and southeast), leishmaniasis is endemic.^12^ The patient's toxic condition, persistence of symptoms, pancytopenia, and travel history to Ardabil, a leishmanial endemic region, led to empirical treatment with amphotericin liposomal. Following the detection of Leishmn's body in a bone marrow sample and the positive serological results, the treatment continued. Microscopy of Bone marrow aspiration is considered a validated diagnostic approach for pediatric patients. Additionally, serological tests including DAT, enzyme‐linked immunosorbent assay, and IFA are considered sensitive for the diagnosis of visceral leishmaniasis among immunocompetent patients.^13^

In the Abdinia et al. study that evaluated pediatric VL in the northwest of Iran, DAT was positive in more than 90% of cases.^13^ A study in Iran has estimated the sensitivity of the observation of Leishman bodies from 35% to 67%.^13^ In this case, we found Leishman bodies in the bone marrow aspirate. The identification of the Leishman‐Donovan bodies was closely related to the experience of laboratorians.

Symptoms and signs associated with VL, include fever, chills, fatigue, and weight loss, loss of appetite, hepatosplenomegaly, and lymphadenopathy. An increase in creatinine occurred in the middle of treatment, and serum therapy and antibiotic adjustment were needed to control it. During hospitalization, the platelet and neutrophil counts decreased. As a result of the decrease in platelets, transfusions were administered, and for persistent neutropenia, G‐CSF 300 mcg for 3 days was prescribed. As the fever did not respond to broad‐spectrum antibiotic therapy, it was decided to begin oral treatment with prednisolone (2 mg/kg/day), with consideration of the Jarisch Herxheimer reaction. A two‐week period later, the patient was discharged due to the clinical improvement. To maintain drug therapy at home, glucantime was administered every 12 h intramuscularly (20 mg/kg of pentavalent antimony, which is equal to 75 mg/kg of glucantime), and prednisolone (2 mg/kg/day). The World Health Organization (WHO) recommends 20 mg/kg of pentavalent antimony for systemic treatment, which is 75 mg/kg of glucantime. For patients weighing 20 kilogram, 1–3 five‐milliliter shots of glucantime per 20 kilogram would be prescribed because each five‐milliliter shot contains 425 milligrams of antimony (1.5 grams of glucantime). Leishmaniasis in rural and urban areas generally requires two and 3 weeks of treatment, respectively. Treatment is considered a failure if no signs of recovery have been observed within 4 weeks of treatment; reintroduction of the previous dosage is recommended.^14^

One week later he was readmitted to the hospital with severe anemia, leukopenia, and orchitis. In the past, the patient had arbitrarily stopped taking prednisolone at home. Hb levels did not increase despite the blood transfusion. Nothing was found in the workup for sepsis. Because of the high inflammatory elements and negative sepsis workup, it was diagnosed as an inflammatory syndrome. IVIG along with methylprednisolone was prescribed for 2 days based on the clinical symptoms and laboratory parameters. 4 days later, the patient's HB increased, his fever stopped, and his overall condition improved.

## 4 CONCLUSION

In our patient's case, we were confronted with the rare combination of VL and of epididymo‐orchitis in the immune‐deficient patient. The presence of the patient's toxic condition, the persistence of symptoms, pancytopenia, testicular pain, and swelling along with epididymitis involvement may point towards the diagnosis of leishmaniasis under an appropriate clinical setting, especially in the endemic counties for leishmaniasis. A definitive diagnosis can be given with sampling and microscopy of bone marrow aspiration, and serological tests including DAT, enzyme‐linked immunosorbent assay, and IFA.

## AUTHOR CONTRIBUTIONS

**Shaghayegh Ashraf Talesh:** Conceptualization; investigation; visualization; writing – original draft. **Shima Mahmoudi:** Supervision; validation; writing – review and editing. **Mehdi Mohebali:** Investigation; methodology; writing – review and editing. **Setareh Mamishi:** Conceptualization; data curation; investigation; methodology; supervision; validation; writing – review and editing.

## CONFLICT OF INTEREST STATEMENT

The authors declare that they have no competing interests.

## ETHICS STATEMENT

We confirm that all named authors have read and approved the manuscript. We have considered about how to protect the intellectual property associated with this manuscript.

## CONSENT

The authors confirmed that they had obtained a patient written consent. The parent had given his permission for his images and other clinical data to be included in the manuscript.

## Data Availability

Since no new data were generated or examined in this study, data sharing does not apply to this publication.

## References

[1] Soler‐Palacín P , Margareto C , Llobet P , et al. Chronic granulomatous disease in pediatric patients: 25 years of experience. Allergol Immunopathol (Madr). 2007;35:83‐89. doi:10.1157/13106774 17594870

[2] Roos D . Chronic granulomatous disease. Br Med Bull. 2016;118:50‐63. doi:10.1093/bmb/ldw009 26983962 PMC5127417

[3] Ben‐Ari J , Wolach O , Gavrieli R , Wolach B . Infections associated with chronic granulomatous disease: linking genetics to phenotypic expression. Expert Rev Anti Infect Ther. 2012;10:881‐894. doi:10.1586/eri.12.77 23030328

[4] Martín A , Marques L , Soler‐Palacín P , et al. Visceral leishmaniasis associated hemophagocytic syndrome in patients with chronic granulomatous disease. Pediatr Infect Dis J. 2009;28:753‐754. doi:10.1097/INF.0b013e31819c6f3a 19633526

[5] Glans H , Dotevall L , Söbirk SK , Färnert A , Bradley M . Cutaneous, mucocutaneous and visceral leishmaniasis in Sweden from 1996–2016: a retrospective study of clinical characteristics, treatments and outcomes. BMC Infect Dis. 2018;18:632. doi:10.1186/s12879-018-3539-1 30526519 PMC6286557

[6] Samaranayake N . Chapter 2 – Leishmaniasis. In: Misra G , Srivastava V , eds. Molecular Advancements in Tropical Diseases Drug Discovery. Academic Press; 2020:21‐46.

[7] Herwaldt BL . Leishmaniasis. Lancet. 1999;354:1191‐1199. doi:10.1016/s0140-6736(98)10178-2 10513726

[8] Chappuis F , Sundar S , Hailu A , et al. Visceral leishmaniasis: what are the needs for diagnosis, treatment and control? Nat Rev Microbiol. 2007;5:873‐882. doi:10.1038/nrmicro1748 17938629

[9] Finocchi A , Palma P , di Matteo G , et al. Visceral leishmaniasis revealing chronic granulomatous disease in a child. Int J Immunopathol Pharmacol. 2008;21:739‐743. doi:10.1177/039463200802100330 18831944

[10] Lora MS , Waguespack SG , Moley JF , Walvoord EC . Adrenal ganglioneuromas in children with multiple endocrine neoplasia type 2: a report of two cases. J Clin Endocrinol Metab. 2005;90:4383‐4387. doi:10.1210/jc.2004-2526 15827098

[11] Al‐Ayed M . Visceral Leishmaniasis and chronic granulomatous disease in an infant. Int J Curr Microbiol App Sci. 2015;4:88‐91.

[12] Abdolsalehi MR , Mohebali M , Keshavarz H , Mahmoudi S , Mamishi S . The emergence of Co‐infection of visceral Leishmaniasis in an iranian child with chronic granulomatous disease: a case report. Iran J Parasitol. 2021;16:159‐163. doi:10.18502/ijpa.v16i1.5536 33786058 PMC7988672

[13] Abdinia B , Oliaei‐Motlagh M , Teimouri‐Dereshki A . Pediatric visceral leishmaniasis in northwest of Iran. Medicine (Baltimore). 2016;95(44):e5261. 27858891 10.1097/MD.0000000000005261 PMC5591139

[14] Meeting WECotCotL, World Health Organization . Control of the Leishmaniases: Report of a Meeting of the WHO Expert Committee on the Control of Leishmaniases, Geneva, 22–26 March 2010: World Health Organization.

